# Crystalloid cardioplegia versus cold blood cardioplegia in aortic arch surgery: A noninferiority randomized trial

**DOI:** 10.1016/j.xjon.2026.101578

**Published:** 2026-01-08

**Authors:** Shota Hasegawa, Katsuhiro Yamanaka, Ryo Kawabata, Hironaga Shiraki, Shunya Chomei, Noriko Ohyama, Taishi Inoue, Soichiro Henmi, Hiroaki Takahashi, Kenji Okada

**Affiliations:** Division of Cardiovascular Surgery, Department of Surgery, Kobe University Graduate School of Medicine, Kobe, Japan

**Keywords:** blood cardioplegia, crystalloid cardioplegia, St. Thomas' Hospital Ⅱ solution, aortic arch surgery, myocardial protection

## Abstract

**Background:**

The optimal cardioplegic solution to use during aortic surgery remains unclear. While cold blood cardioplegia (BCP) has metabolic advantages, crystalloid cardioplegia (CCP) offers practical benefits. This trial investigated whether cold CCP is noninferior to cold BCP in preserving postoperative left ventricular ejection fraction (LVEF).

**Methods:**

In this single-center, patient- and assessor-blinded, parallel-group noninferiority randomized trial, 52 adult patients undergoing elective aortic arch replacement were randomized 1:1 to receive cold BCP or CCP. The primary endpoint was LVEF on postoperative day 7. The noninferiority margin was set at −7%. Secondary endpoints included change in LVEF, mortality, low-output syndrome, myocardial infarction (MI), creatine kinase MB isotype (CK-MB) release, left ventricular diastolic dysfunction, right ventricular systolic dysfunction, stroke, atrial fibrillation, pacemaker implantation, mediastinal drainage, reexploration for bleeding, and acute kidney injury.

**Results:**

The median aortic cross-clamp time was 96 minutes. Baseline LVEF was similar in the 2 groups (BCP, 61.1 ± 5%; CCP, 61.7 ± 5%; *P* = .66). The mean difference in postoperative LVEF (CCP – BCP) was 1.23%, with a 95% confidence interval of −5.48% to 2.69%, exceeding the noninferiority margin and confirming noninferiority (*P* = .0041). Peak CK-MB levels were comparable in the 2 groups (BCP, 35.0 ± 15 U/L; CCP, 41.6 ± 22 U/L; *P* = .22), although levels at 7 hours and 24 hours were lower in the BCP group (*P* = .034 and .046, respectively). One in-hospital death occurred in the BCP group, and 1 case of low output syndrome occurred in the CCP group. Postoperative MI occurred in 4 patients (2 per group), with no significant differences in other secondary endpoints.

**Conclusions:**

Cold CCP appeared to be noninferior to cold BCP for myocardial protection in elective aortic arch replacement, as suggested by comparable postoperative LVEF.

**Figure:**
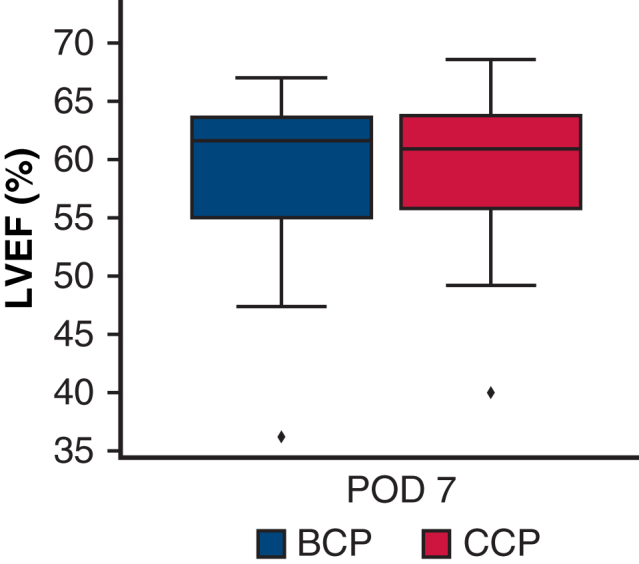
Left ventricular ejection fraction on postoperative day 7. The *box* represents the interquartile range, the *line* inside indicates the median, and the whiskers represent the minimum and maximum values excluding outliers. Symbols outside the whiskers represent outliers.

**Central Message**

Cold crystalloid cardioplegia was noninferior to cold blood cardioplegia for preserving postoperative left ventricular ejection fraction after elective aortic arch replacement with prolonged ischemia.

**Perspective**

In this patient- and assessor-blinded noninferiority trial (n = 50) of elective aortic arch replacement, cold crystalloid cardioplegia preserved left ventricular ejection fraction on postoperative day 7 as effectively as cold blood cardioplegia. This is the first randomized study of prolonged ischemia for complex aortic surgery, contributing important evidence to guide myocardial protection strategies.

Myocardial protection is a crucial component of cardiovascular surgery and has been progressively refined over the past 70 years. Cold blood cardioplegia (BCP) has theoretical advantages^1^^,^^2^ and has been compared with crystalloid cardioplegia (CCP) in trials and meta-analyses to identify the optimal strategy.^3^, ^4^, ^5^ Some reports have suggested that BCP is associated with lower myocardial enzyme release and reduced rates of postoperative myocardial infarction (MI) and low cardiac output syndrome (LCOS).^3^^,^^4^ Despite these findings, most prior trials focused on coronary artery bypass grafting (CABG), which involves relatively short aortic cross-clamp times. Consequently, current evidence remains insufficient for determining the clinical superiority of any cardioplegic solution, particularly in aortic surgery requiring prolonged myocardial ischemia.^6^

CCP offers practical simplicity, requiring no complex mixing and enabling a clearer surgical field. In particular, St. Thomas’ Hospital II solution allows for longer dosing intervals compared to BCP, minimizing procedural interruptions.^7^, ^8^, ^9^

In this study, we conducted a randomized controlled trial comparing cold BCP and cold CCP in patients undergoing an aortic arch replacement, a relatively standardized procedure with consistent and sufficiently prolonged myocardial ischemic times. We hypothesized that CCP would provide noninferior preservation of left ventricular (LV) systolic function compared to BCP.

## Methods

### Trial Design

**Figure E1:**
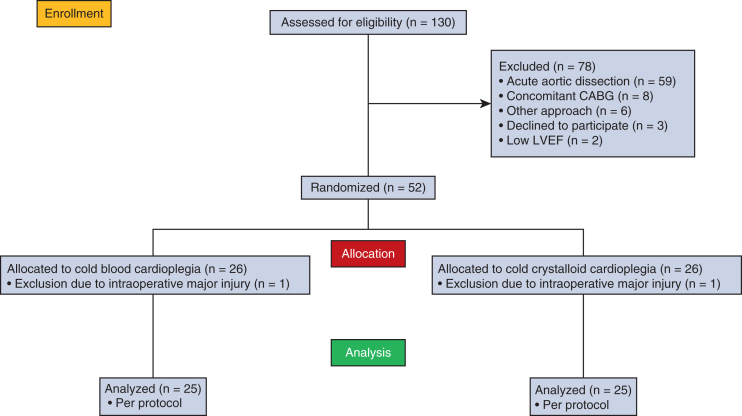
The trial flow chart. *BCP*, Blood cardioplegia; *CCP*, crystalloid cardioplegia.

This single-center, prospective, patient- and assessor-blinded, parallel-group noninferiority randomized trial was conducted between September 2022 and November 2024 in Japan to compare cold BCP and CCP (Figure E1). All subjects provided written informed consent for the publication of their study data, and the study was approved by the local Ethics Committee (reference C220002, approved June 14, 2022) and was registered with the Japan Registry of Clinical Trials (jRCTs051220064; https://jrct.mhlw.go.jp/latest-detail/jRCTs051220064). This randomized controlled trial was conducted and reported in accordance with the CONSORT 2010 statement.

Patients age ≥18 years undergoing elective an aortic arch replacement via median sternotomy at Kobe University Hospital were eligible. Patients with coronary artery stenosis (≥75%) necessitating intervention, as well as those with a left ventricular ejection fraction (LVEF) ≤50%, were excluded. Concomitant procedures were performed as needed. Initially limited to total arch replacement, the protocol was amended in November 2022 to include hemiarch and partial arch replacement, owing to concerns about insufficient enrollment within the planned timeframe.

### Randomization

Participants were allocated at random to either the cold BCP group or cold CCP group prior to the day of surgery, using a web-based electronic data capture system. The randomization was performed at a 1:1 ratio using the permuted block method with a block size of 4.

### Echocardiography

All echocardiographic analyses were performed by at least 2 blinded cardiologists or echocardiographers. The Simpson biplane method of discs was used to calculate LVEF.^10^ Regional wall motion abnormality was assessed qualitatively by transthoracic echocardiography (TEE).

### Anesthesia Protocol

The anesthesia protocol was standardized. Induction used propofol 0.5 to 2 mg/kg and/or midazolam 1 to 5 mg, followed by rocuronium 0.6 mg/kg, fentanyl 1 to 5 μg/kg, and/or remifentanil 0.1 to 0.5 μg/kg/minute. For maintenance, sevoflurane or propofol was combined with remifentanil, fentanyl, and rocuronium.

### Surgical Technique

Aortic arch replacement was performed via median sternotomy under deep hypothermic circulatory arrest (tympanic 20°C-23°C; rectal <30 °C) with antegrade selective cerebral perfusion, as described previously^11^^,^^12^ (Video 1). Cardiopulmonary bypass (CPB) was established with bicaval drainage and arterial perfusion through the ascending aorta in most cases. After systemic cooling, the aortic arch was opened once the tympanic temperature decreased to 23 °C and the rectal temperature was below 30 °C. Brief retrograde cerebral perfusion through the superior vena cava was applied during the initiation of circulatory arrest, and selective antegrade cerebral perfusion was then initiated through balloon-tipped cannulas placed in the brachiocephalic, left carotid, and left subclavian arteries. Distal anastomosis was performed first under circulatory arrest, followed by reinstitution of lower body perfusion and rewarming to 33 °C. The proximal anastomosis was then completed, and coronary reperfusion was initiated. Arch vessels were reconstructed sequentially under continued selective antegrade cerebral perfusion. In patients needing aortic root replacement, arch branches were reconstructed after distal anastomosis, followed by root replacement and coronary reperfusion.

For most patients, the ascending aorta was clamped simultaneously with the circulatory arrest, and cardioplegia was delivered anterogradely for the initial dose. In cases of true aneurysms with atherosclerotic plaque, dissected aneurysms with thrombus in the false lumen, or severe aortic insufficiency, retrograde delivery was used for the initial administration instead. Retrograde delivery was routinely combined for all cases, while antegrade delivery was performed initially whenever technically feasible. After opening the aorta, both coronary ostia were inspected. If retrograde return was found to be inadequate, selective antegrade cardioplegia also would have been administered directly into the coronary ostia; however, no such cases occurred in this series. In patients undergoing aortic valve replacement or root replacement, selective antegrade cardioplegia—defined as direct infusion into the left and right coronary ostia—was also used. Cardioplegia was administered according to the assigned protocol.

### Myocardial Protection

One of the 2 cardioplegic solutions was used for myocardial protection. The compositions of the BCP and CCP solutions are summarized in Table 1.

**Table 1:** Electrolyte and lidocaine concentrations in the cardioplegic solutions

Parameter	BCP	CCP
Na^+^, mmol/L	122	120
K^+^, mmol/L	19	16
Mg^2+^, mmol/L	8	16
Ca^2+^, mmol/L	0.4-0.5	1.2
Lidocaine, mg/L	10	200

*BCP*, Blood cardioplegia; *CCP*, crystalloid cardioplegia.

### BCP Protocol

The cold BCP was a mixture of autologous blood from the cardiopulmonary circuit and the crystalloid in a 4:1 ratio (blood/crystalloid). The composition of the crystalloid was 500 mL of normal saline, 150 mL of trihydroxy methyl aminomethane, 60 mL of KCl 60 mEq/L KCl, 50 mL of citrate-phosphate-dextrose-adenine, 40 mL of magnesium sulfate 1 mEq/L, 10 mL of dextrose 50% in water, and 2 mL of lidocaine 1%. This solution was mixed with autologous blood from the pump circuit in a 4:1 ratio (blood/solution). The dosage was 10 mL/kg (minimal dose of 500 mL). The solution temperature was 12 °C. After the initial delivery, additional doses were given every 35 minutes or when any electrical activity was observed. Before aortic declamping, 500 mL of 37 °C solution mixed 16:1 with blood and the crystalloid was injected via either an antegrade or a retrograde route as terminal warm BCP.

### CCP Protocol

We administered a crystalloid solution composed of 10 mL/kg of St. Thomas’ Hospital II solution (Miotector; Fuso Pharmaceutical Industries) containing 2 mg of lidocaine per 10 mL/kg, at 4 °C. The solution was delivered anterogradely at 60 minutes and retrogradely at 30 minutes.

### Study Outcomes

The primary outcome was postoperative LV systolic function, as measured by LVEF via TTE on postoperative day 7. Assessment on postoperative day 7 was chosen to minimize the influence of transient myocardial stunning and to reflect stabilized cardiac function. Secondary outcomes included percentage change in LVEF, in-hospital death, LCOS, MI, creatine kinase MB isotype (CK-MB) levels, new-onset LV diastolic or right ventricular systolic dysfunction, atrial fibrillation, pacemaker implantation, defibrillation after reperfusion, reexploration for bleeding, mediastinal drainage at 24 hours postoperatively, and acute kidney injury (AKI).

### Definitions of Outcomes

Blood samples were collected preoperatively and at 7, 24, 48, and 72 hours postoperatively, based on enzyme release kinetics after myocardial ischemia.^13^ Myocardial injury was assessed based on the highest CK-MB levels. Twelve-lead electrocardiograms were recorded at postoperative 1, 24, 48, and 72 hours and day 7 and compared with preoperative electrocardiograms. MI was defined by ≥2 of the following: (1) CK-MB ≥100 μg/L or CK-MB/CK ≥10%, (2) new Q wave ≥0.03 seconds, and (3) new hypokinetic or akinetic region on TTE. AKI was defined as a ≥1.5-fold or ≥0.3 mg/dL increase in creatinine.^14^ LCOS was defined as requiring dobutamine ≥10 μg/kg/minute for >30 minutes, epinephrine ≥0.1 μg/kg/minute, or intra-aortic balloon pump implantation. The percentage change in LVEF was calculated as: (post - pre)/pre × 100, and multiplied by 100. LV diastolic dysfunction was defined by either (1) mitral inflow early diastolic velocity (E wave) to atrial contraction velocity ratio ≥1.5 or (2) E wave deceleration time ≤140 ms.^15^ Right ventricular systolic dysfunction was defined as tricuspid annular plane systolic excursion <17 mm. Atrial fibrillation was defined as paroxysmal episodes ≥30 seconds or persistent episodes within 48 hours. Ventricular arrhythmia after declamping was defined as requiring defibrillation within 48 hours, corresponding to the early postoperative period (within 2-3 days) commonly adopted in previous randomized trials.

### Statistical Analysis

The sample size was calculated based on 80% power and a one-sided significance level of 0.025, assuming an expected between-group difference of 0% and a noninferiority margin of −7%. The expected mean postoperative LVEF was set at approximately 60%, based on previously published data derived from larger patient series.^16^ The standard deviation was derived from our institutional data. Among 333 patients with preoperative LVEF ≥50%, postoperative LVEF was available in 111 patients, with a mean of 62.5% and a standard deviation of 8.42%. Thus, the literature-based mean was used for estimating the expected postoperative value, whereas the institutional standard deviation was used to provide a realistic estimate of variability in our population.

The noninferiority margin of −7% was set so that the lower bound of the acceptable range would not fall below 52%, which is the lower limit of normal systolic LV function.^17^ Based on these assumptions, 24 patients per group were required; allowing for potential dropouts, the final planned sample size was 26 patients per group (52 total).

The sample size was calculated based solely on the primary endpoint, and thus the precision of the evaluations for the secondary outcomes—including infrequent clinical events—may be insufficient and should be interpreted with caution. The assumptions and sample-size calculation were validated in consultation with a biostatistician to confirm the adequacy of the noninferiority design.

The primary analysis was conducted on a per-protocol population, which included all patients who received the allocated cardioplegia and had assessable primary outcome data, excluding patients with major intraoperative complications. Categorical variables were expressed as absolute numbers and percentages, and continuous variables were expressed as mean value with standard deviation (SD) or median value with interquartile range (IQR). For the primary outcome of postoperative LVEF, an unpaired *t* test was used to compare the 2 groups, as the main study hypothesis concerned between-group noninferiority rather than within-patient change. Additionally, paired analyses comparing preoperative and postoperative LVEF within each group were performed as a supplementary analysis to confirm the consistency of the results. For other outcomes, the Fisher exact test was applied to categorical variables, while continuous variables were analyzed using either an unpaired *t* test or the Mann-Whitney *U* test, as appropriate. For the primary outcome, noninferiority was concluded if the lower limit of the 95% confidence interval (CI) for the between-group difference exceeded the noninferiority margin of −7. All *P* values tested the null hypothesis of no difference. All statistical calculations were performed using JMP Pro 15.0.0 (SAS Institute). Secondary endpoints were not adjusted for multiplicity correction.

## Results

### Patient Population

Between September 2022 and November 2024, a total of 52 patients were enrolled and randomized to the BCP group (n = 26) or the CCP group (n = 26). Two patients (1 in each group) were excluded from the analysis because of major intraoperative injuries. The remaining 50 patients were included in the per-protocol analysis (Figure E1).

Patient characteristics were similar in the 2 groups (Table 2). The median age of the entire cohort was 75 years (IQR, 65-81 years), and 86.5% of the patients had a true aneurysm. One patient had a history of prior median sternotomy; however, no patients had a history of cardiac or great vessel surgery. The median EuroSCORE Ⅱ and Japan score for the overall cohort were 2.57% (IQR, 1.74%-4.45%) and 2.95% (IQR, 2.28%-5.48%), respectively. The mean preoperative LVEF was 61.4 ± 5% overall, with no significant difference between the 2 groups (*P* = .664).

**Table 2:** Patient characteristics

Characteristic	BCP group	CCP group	*P* value
Age, y, median [IQR]	75 [68-82]	76 [67-81]	>.99
Male sex, n (%)	14 (56)	14 (56)	>.99
True aneurysm, n (%)	21 (84)	22 (88)	>.99
Dissected aneurysm, n (%)	4 (16)	3 (12)	>.99
AS, n (%)	2 (8)	1 (4)	>.99
AR, n (%)	4 (16)	3 (12)	>.99
History, n (%)			
Hypertension	19 (76)	22 (88)	.464
Dyslipidemia	9 (36)	14 (56)	.256
Diabetes mellitus	1 (4)	3 (12)	.609
CKD	4 (16)	6 (24)	.725
COPD	4 (16)	2 (8)	.667
Smoking	14 (56)	15 (60)	.567
Atrial fibrillation	3 (12)	5 (20)	.702
Cerebral infarction	3 (12)	1 (4)	.609
PCI	2 (8)	1 (4)	>.99
Median sternotomy	1 (4)	0	>.99
Treatment, n (%)			
Anticoagulants	2 (8)	4 (16)	.667
Antiplatelets	7 (28)	5 (20)	.742
Steroids	0	4 (16)	.110
Beta blockers	6 (24)	9 (36)	.538
Calcium channel blockers	14 (56)	19 (76)	.232
EuroSCORE II, median [IQR]	2.8 [1.9-4.8]	2.3 [1.3-4.1]	.290
Japan score, median [IQR]	3.2 [2.2-5.5]	2.7 [2.3-5.8]	.970
LVEF, %, mean ± SD	61.1 ± 5	61.7 ± 5	.664

*BCP*, Blood cardioplegia; *CCP*, crystalloid cardioplegia; *IQR*, interquartile range; *AS*, aortic stenosis; *AR*, aortic regurgitation; *CKD*, chronic kidney disease; *COPD*, chronic obstructive pulmonary disease; *PCI*, percutaneous coronary intervention; *LVEF*, left ventricular ejection fraction; *SD*, standard deviation.

### Operative Data

Thirty-six patients (72%) underwent total arch replacement (Table 3). The BCP group had more aortic valve replacement surgeries, which was associated with more selective antegrade cardioplegia. Of the 7 patients who underwent aortic valve replacement, 6 had been diagnosed with moderate or greater aortic insufficiency. The median aortic cross-clamp time for all patients was 96 minutes (IQR, 79 to 124 minutes).

**Table 3:** Operative details

Variable	BCP group	CCP group	*P* value
Range of replacement, n (%)			.614
Zone 0	1 (4)	1 (4)	
Zone 1	4 (16)	2 (8)	
Zone 2	4 (16)	2 (8)	
Zone 3	16 (64)	20 (80)	
Concomitant procedure, n (%)			
AVR	6 (24)	1 (4)	.0983
Bentall	1 (4)	1 (4)	>.99
VSRR	1 (4)	1 (4)	>.99
Operative time, min, median [IQR]	365 [339-435]	358 [335-391]	.523
CPB time, min, median [IQR]	225 [182-244]	211 [189-223]	.836
Aortic cross-clamp time, min, median [IQR]	112 [91-132]	91 [75-106]	.450
Selective cerebral perfusion time, min, median [IQR]	74 [63-88]	78 [65-92]	.583
Lowest rectal temperature, °C, mean ± SD	23.1 ± 0.9	23.3 ± 1.0	.420
Delivery route, n (%)			
Antegrade + retrograde	22 (88)	21 (84)	>.99
Retrograde only	3 (12)	4 (16)	>.99
Antegrade selective	6 (24)	2 (8)	.247
Defibrillation after declamping, n (%)	3 (12)	2 (8)	>.99
Intraoperative hemoglobin nadir, g/dL, mean ± SD	6.99 ± 0.90	6.83 ± 0.81	.523
Intraoperative transfusion, units, median [IQR]			
Red blood cells	8 [6-12]	8 [6-10]	.575
Fresh frozen plasma	6 [6-10]	6 [4-10]	.548
Platelets	10 [0-20]	10 [0-15]	>.99
Intraoperative blood loss, mL, median [IQR]	621 [356-1000]	750 [363-1049]	.472

*BCP*, Blood cardioplegia; *CCP*, crystalloid cardioplegia; *AVR*, aortic valve replacement; *VSRR*, valve-sparing root replacement; *IQR*, interquartile range; *CPB*, cardiopulmonary bypass; *SD*, standard deviation.

### Primary Outcome

**Figure 1:**
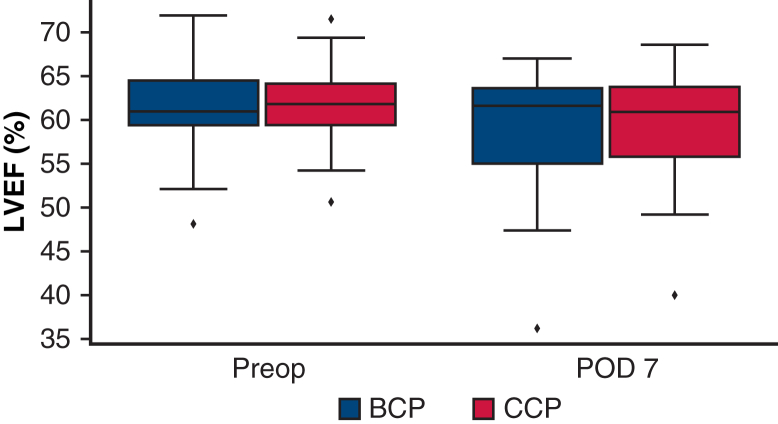
Comparison of left ventricular ejection fraction (LVEF) preoperatively and on postoperative day 7 between the blood cardioplegia (BCP; *blue*) group and crystalloid cardioplegia (CCP; *red*) groups. Data are presented as boxplots; the *box* represents the interquartile range (25th and 75th percentiles), the *line* inside the *box* indicates the median, and the whiskers represent the minimum and maximum values excluding outliers. Symbols outside the whiskers represent outliers. *POD*, Postoperative day.

The mean postoperative LVEF was 58.3 ± 7% in the BCP group and 59.5 ± 7% in the CCP group (Table 4, Figure 1). The mean difference in postoperative LVEF between the CCP and BCP groups was 1.23% (95% CI, −2.89% to 4.12%; *P* = .540). Because the lower bound of the 95% CI was above the predefined noninferiority margin of −7%, CCP was considered noninferior to BCP (95% CI: −5.48% to 2.69%; *P* = .0041). This result was consistent in both the per-protocol and intention-to-treat analyses.

**Table 4:** Postoperative outcomes

Outcome	BCP group	CCP group	*P* value
Postoperative LVEF, %, mean ± SD	58.3 ± 7	59.5 ± 7	.540
% change in LVEF, mean ± SD	−3.7 ± 11	−4.7 ± 14	.779
LV diastolic dysfunction, n (%)	4 (16)	1 (4)	.349
RV systolic dysfunction, n (%)	14 (56)	11 (44)	.572
CK-MB, U/L, median [IQR]			
1 h	32.0 [27.0-41.0]	36.0 [32.0-46.0]	.282
7 h	20.0 [14.0-29.0]	28.0 [23.0-37.0]	.0344
24 h	8.0 [6.0-11.0]	11.0 [9.0-15.0]	.046
48 h	5.0 [3.0-6.0]	4.0 [3.0-6.0]	.377
72 h	3.0 [3.0-5.0]	3.0 [3.0-4.0]	.650
In-hospital death, n (%)	1 (4)	0	>.99
LCOS, n (%)	0	1 (4)	>.99
Postoperative MI, n (%)	2 (8)	2 (8)	>.99
Stroke, n (%)	1 (4)	0	>.99
Mechanical ventilation time, h, mean ± SD	31.6 ± 8	14.7 ± 7	.1
Transfusion, mL, median [IQR]	760 [480-1390]	760 [360-1400]	>.99
Mediastinal drainage 24 h postoperatively, mL, median [IQR]	930 [671-1098]	1010 [795-1275]	.94
Reexploration for bleeding, n (%)	0	0	>.99
New-onset atrial fibrillation, n (%)	4 (16)	9 (36)	.1
Pacemaker implantation, n (%)	0	0	>.99
Acute kidney injury, n (%)	4 (16)	6 (24)	.725

The sample size was calculated based on the analysis of LVEF, and thus the precision of the evaluations for the secondary outcomes—including clinical events—may be insufficient and should be interpreted with caution.

*BCP*, Blood cardioplegia; *CCP*, crystalloid cardioplegia; *SD*, standard deviation; *LVEF*, left ventricular ejection fraction; *LV*, left ventricular; *RV*, right ventricular; *CK-MB*, creatine kinase MB isotype; *IQR*, interquartile range; *LCOS*, low cardiac output syndrome; *MI*, myocardial infarction.

### Secondary Outcomes

There was 1 in-hospital death in the BCP group, due to brainstem infarction and pneumonia (Table 4). There was one case of postoperative LCOS: a 50-year-old woman in the CCP group who underwent Bentall and total arch replacement. After 290 minutes of aortic cross-clamping, intra-aortic balloon pump support was required. The peak CK-MB level was 55 U/L, which did not meet the criteria for postoperative MI.

Postoperative MI, defined by CK-MB/CK >10% and newly detected postoperative wall motion abnormality, occurred in 4 patients (2 per group; *P* > .99). In all cases, the regions of wall motion abnormality were limited and did not affect overall LV function. Aortic cross-clamp times ranged from 90 to 162 minutes. In all 4 of these patients, MI had no clinically significant impact on the postoperative course.

**Figure 2:**
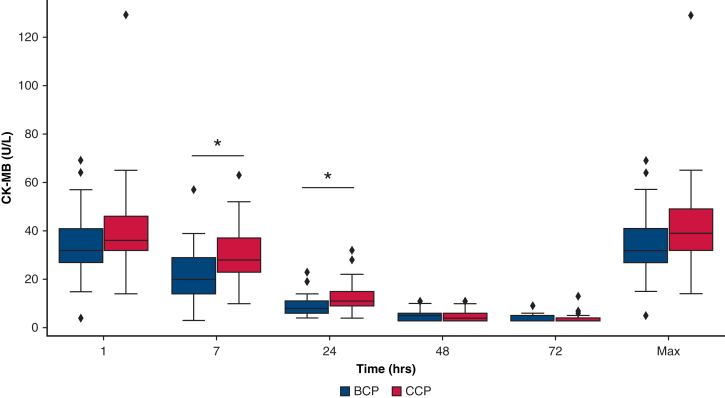
Postoperative creatine kinase MB isotype (CK-MB) levels at 1, 7, 24, 48, 72 hours, and the peak values in the blood cardioplegia (BCP, *blue*) and crystalloid cardioplegia (CCP; *red*) groups. Data are presented as boxplots; the *box* represents the interquartile range (25th and 75th percentiles), the *line* inside the *box* indicates the median, and the whiskers represent the minimum and maximum values excluding outliers. Asterisks indicate statistically significant differences (*P* < .05). Symbols outside the whiskers represent outliers.

The highest CK-MB levels were found at 1 hour after surgery in both groups (Table 4, Figure 2). Although CK-MB levels at 7 and 24 hours postoperatively were significantly lower in the BCP group (*P* = .031 and .046, respectively), there was no significant difference in the maximum CK-MB level between the 2 groups (*P* = .218) (Table 4).

**Figure 3:**
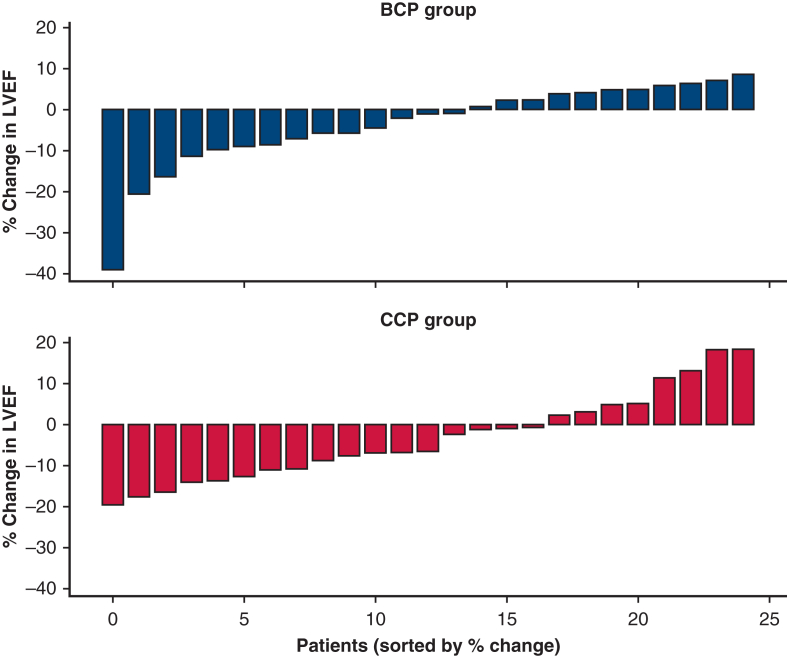
Waterfall plot of percentage change in left ventricular ejection fraction from baseline to day 7. *BCP*, Blood cardioplegia; *CCP*, crystalloid cardioplegia.

The percentage change in LVEF on postoperative day 7 was −4.2 ± 12.6% in the entire cohort, with no significant difference observed between the 2 groups (*P* = .779) (Table 4, Figure 3).

There were no significant differences between the groups in postoperative LV diastolic dysfunction, right ventricular systolic dysfunction, mechanical ventilation time, transfusion volume, drainage volume, reexploration for bleeding, new-onset atrial fibrillation, or AKI (Table 4).

## Discussion

This trial suggests that CCP is not inferior to cold BCP in preserving postoperative LVEF. Secondary outcomes showed no apparent differences; however, the study was underpowered to detect differences in infrequent clinical events. Although many randomized trials have compared BCP and CCP, most involved CABG with short cross-clamp times; the mean across 36 trials was 62.8 ± 20 minutes (range, 34-107 minutes). In contrast, this study is the first randomized trial with more than 50 patients undergoing arch replacement, 72% of whom underwent a total arch replacement, with longer cross-clamp times and homogeneous characteristics. The 2024 European Association for Cardio-Thoracic Surgery/Society of Thoracic Surgeons guidelines state that no cardioplegic solution has proven superior in aortic surgery^6^; therefore, the findings from this study provide valuable clinical evidence.

Cardioplegia delivery followed a standardized institutional protocol applied uniformly in both groups, minimizing the risk of bias related to antegrade, selective antegrade, or retrograde administration. Although concomitant aortic valve replacement was more frequent in the BCP group, cross-clamp and CPB times were similar in the 2 groups. Exploratory subgroup analyses stratified by concomitant aortic valve replacement and by the extent of arch repair revealed no qualitative interaction with the allocated cardioplegic solutions, although these analyses were underpowered and should be interpreted with caution (Table E1).

Postoperative LVEF was confirmed to be noninferior in the CCP group compared to the BCP group. Our results differ from the findings of previous randomized trials that compared postoperative LV function.^18^, ^19^, ^20^ Several previous trials have reported that the use of BCP was associated with less postoperative decline in the LV stroke work index compared to CCP. This contrast may reflect differences in patient populations and evaluation timing; most previous trials focused solely on patients undergoing CABG and assessed cardiac function within several hours to 2 days postoperatively. In our study, echocardiographic evaluation was performed on postoperative day 7, when the stunned myocardium was expected to have recovered.^21^

The incidence of postoperative MI was higher in the present study compared to that reported in previous randomized trials, which ranged from 1.2% to 3.1% in BCP groups and from 2.4% to 3.7% in CCP groups.^3^, ^4^, ^5^ The higher incidence in our study might be related to our broader diagnostic criteria, which included echocardiographic findings. This approach was not commonly adopted in previous trials, which likely increased detection sensitivity. Notably, none of the MI cases had a clinically relevant impact on postoperative course.

The BCP group showed significantly lower enzyme release at 7 and 24 hours postoperatively, consistent with prior reports.^3^ Although the early enzyme release showed a statistically significant difference in the CCP group, the values did not reach a level associated with clinically significant myocardial injury. The reduced enzyme release in the BCP group may reflect abundant oxygen delivery, reducing ischemic debt and supporting aerobic metabolism.^2^

At our institution, lidocaine is routinely added to the cardioplegic solution. Lidocaine concentrations of 100 to 130 mg/L have been associated with a reduced incidence of ventricular fibrillation following coronary reperfusion,^16^^,^^22^ while 500 mg/L has been associated with an increased risk of high-grade atrioventricular block.^23^ In the present study, although a relatively high lidocaine concentration was used, no patients required pacemaker implantation.

Beyond comparable myocardial protection, CCP offers additional practical benefits. Its preparation does not require complex mixing, which reduces the risk of human error. This simplicity may contribute to improved reproducibility and patient safety, especially in high-volume or resource-limited settings.

### Limitations

This single-center trial had a relatively small sample size. Because the sample size calculation was based solely on the analysis of LVEF, the precision for evaluating secondary outcomes—including uncommon clinical events—was inherently limited. Accordingly, interpretation of these secondary endpoints requires caution. This study included heterogeneity in operative procedures, including variations in the extent of arch repair and differences in the proportion of concomitant aortic valve replacement between groups; however, cross-clamp and CPB times were comparable in the 2 groups owing to a standardized operative strategy, and the trial was not powered to assess treatment effects within surgical subgroups. In addition, surgeons were not blinded to the assigned cardioplegia, and outcome assessments were restricted to the early postoperative period.

## Conclusions

CCP appeared to be noninferior to cold BCP for myocardial protection in elective aortic arch replacement, as suggested by comparable postoperative LV function.

## Conflict of Interest Statement

The authors reported no conflicts of interest.

The *Journal* policy requires editors and reviewers to disclose conflicts of interest and to decline handling or reviewing manuscripts for which they may have a conflict of interest. The editors and reviewers of this article have no conflicts of interest.

## Figures and Tables

**Table E1:** Subgroup analysis of postoperative LVEF according to surgical variables

Subgroup	BCP, n	Postoperative LVEF, %, mean ± SD	CCP, n	Postoperative LVEF, %, mean ± SD	Difference, BCP – CCP (95% CI)	Qualitative interaction
Concomitant procedure						
AVR (−)	19	59.9 ± 5.3	24	59.2 ± 6.7	0.6 (−3.1 to 4.3)	No
AVR (+)	6	53.4 ± 11.0	1	67.0	−13.6 (N/A)	N/A
Range of replacements						
Zone 3	16	59.9 ± 5.6	20	58.6 ± 6.8	1.3 (−2.9 to 5.5)	No
Zone 0-2	9	55.5 ± 9.6	5	63.4 ± 5.2	−7.9 (−16.5 to 0.7)	No

This subgroup analysis was exploratory and not powered for formal interaction testing. Qualitative interaction was not observed in any subgroup. Although point estimates differed in direction between Zone 0-2 and Zone 3 repair, confidence intervals were wide and did not support a meaningful qualitative interaction.

*BCP*, Blood cardioplegia; *LVEF*, left ventricular ejection fraction; *SD*, standard deviation; *CCP*, crystalloid cardioplegia; *AVR*, aortic valve replacement; *N/A*, not applicable.

## Supplementary Data

Video 1 Total arch replacement for a distal aortic arch aneurysm with selective antegrade cerebral perfusion under deep hypothermic circulatory arrest. Video available at: https://www.jtcvs.org/article/S2666-2736(26)00001-X/fulltext.
