# Supplementary figures and images for: Crystalloid cardioplegia versus cold blood cardioplegia in aortic arch surgery: A noninferiority randomized trial
Source: JTCVS Open. 2026 Jan 8;30:101578. doi: 10.1016/j.xjon.2026.101578 (PMC13131149; doi:10.1016/j.xjon.2026.101578)

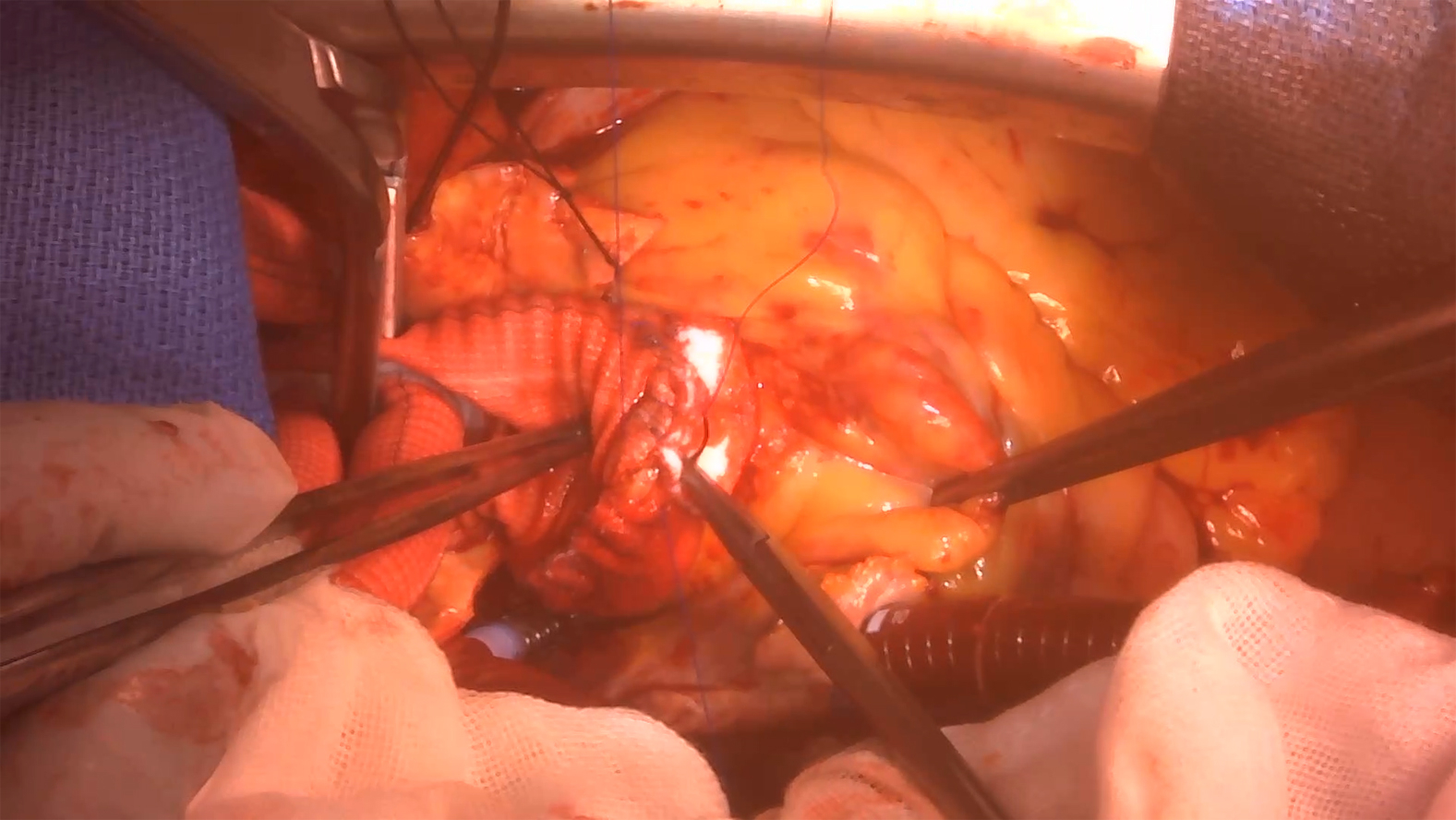

Supplement: Video 1 — Total arch replacement for a distal aortic arch aneurysm with selective antegrade cerebral perfusion under deep hypothermic circulatory arrest. Video available at: https://www.jtcvs.org/article/S2666-2736(26)00001-X/fulltext. [file fx2.jpg]
